# The Association between Macrosomia and Amino Acids’ Levels in Maternal and Cord Sera: A Case-Control Study

**DOI:** 10.3390/nu15153440

**Published:** 2023-08-03

**Authors:** Xinxin Xing, Yifan Duan, Ye Wang, Jie Wang, Zhenyu Yang, Lijun Shao, Lin Li, Jianqiang Lai

**Affiliations:** 1 National Institute for Nutrition and Health, Chinese Center for Disease Control and Prevention, Beijing 100050, China; tao280441@163.com (X.X.); duanyf@ninh.chinacdc.cn (Y.D.); wangye@ninh.chinacdc.cn (Y.W.); wangjie@ninh.chinacdc.cn (J.W.); yangzy@ninh.chinacdc.cn (Z.Y.); 2 Beijing Health Bio Technology Co., Ltd., Beijing 102200, China; lijun.shao@haosibio.com (L.S.); linli@haosibio.com (L.L.)

**Keywords:** macrosomia, amino acids, maternal blood, cord blood, case-control study

## Abstract

This study aims to explore the relationship between macrosomia and amino acids in maternal and cord sera. Methods: In the case-control study, 78 pairs of mothers and newborns were recruited from December 2016 to November 2019. Participants were divided into the macrosomia group (BW ≥ 4000 g, *n* = 39) and the control group (BW between 2500 g and 3999 g, *n* = 39) according to the birth weight (BW) of newborns. Maternal vein blood samples were collected before delivery and cord vein blood samples were collected after birth. The levels of amino acids in maternal and cord sera were measured by liquid chromatography and mass spectrometry (LC-MS/MS) in the year 2021. The difference in amino acid levels in maternal and cord sera between the two groups was compared, and the contribution of each amino acid to the difference between the two groups was analyzed. Unconditional logistic regression analysis was used to test the relationship between macrosomia and amino acids. Results: In maternal serum during the antepartum, the levels of asparagine, glutamine, methionine, alanine, and threonine in the macrosomia group were higher but arginine was lower than that in the control group (*p* < 0.05). In cord serum, the levels of lysine, histidine, phenylalanine, arginine, tryptophan, valine, isoleucine, glutamate, tyrosine, and total essential amino acid (EAA) in the macrosomia group were lower while glutamine was higher than that in the control group (*p* < 0.05). The ratios of EAA, valine, threonine, methionine, tryptophan, and alanine in maternal serum to those in cord serum were higher, while the ratio of glutamine was lower in the macrosomia group (*p* < 0.05). Arginine and threonine in maternal serum and glutamate, glutamine, and histidine in cord serum were associated with macrosomia (*p* < 0.05). Conclusion: Most of the amino acid levels in the maternal sera of the macrosomia group are higher than those in the control group, while most of the amino acids’ levels in the cord sera of the macrosomia group are lower than those in the control group. The ratios of some amino acids in maternal serum to those in cord serum were different between the two groups. Arginine and threonine in maternal serum and glutamate, glutamine, and histidine in cord serum are closely related to macrosomia.

## 1. Introduction

Macrosomia refers to newborns whose birth weight reach or exceed 4000 g [1]. With the development of the economy, the living conditions of Chinese residents are constantly improving, and the incidence rate of macrosomia has also been increasing gradually. In 1995, the incidence rate of macrosomia in China was 6.0% [2], which increased to 6.5% in 2006 [3], 7.3% in 2011 [4], and 8.7% in 2014 [5]. Macrosomia has adverse effects on maternal and infant health in the short and long term. It can increase the probability of perineal trauma, postpartum hemorrhage, cesarean section, and shoulder dystocia [6,7]. Moreover, it can increase perinatal mortality, overweightness, obesity, and metabolic diseases, such as diabetes and hypertension in adulthood [8,9,10]. In addition to physical effects, a high birth weight may also affect the mental health of adolescents [11].

Fetal weight gain is influenced by both genetic predisposition and environmental factors. Environmental factors mainly include the supply and regulation of nutrients [12,13]. Amino acids are important basic substances for the synthesis of structural proteins, enzymes, and protein hormones during fetal growth and development. In addition to playing an important role in nutrient metabolism, amino acids also reflect the nutritional status of the body to some extent [14,15].

Current studies on the association between macrosomia and amino acids in maternal and cord sera are limited. Shang et al. found that up-regulation of placental amino acid transporters may contribute to more macrosomia in women with gestational diabetes mellitus (GDM) and activation of insulin-like growth factor 1 (IGF-I), and mTOR signaling pathways might be involved in this effect [16]. Kuruvilla et al. found that the number of system A (mainly transport of neutral amino acids, such as alanine and glycine) transporters per milligram of membrane protein in the placental microvillous membrane is selectively reduced in diabetic pregnancies associated with macrosomia, while other transport systems were unchanged [17]. The study by Wen showed that increased glutamate and phenylalanine concentrations in the first and second trimesters were associated with an increased risk of macrosomia [18]. The dependent variables of previous studies mostly were birth weight (BW), small for gestational age (SGA), or intrauterine growth restriction (IUGR). In addition, only amino acids in maternal blood or in cord blood were analyzed [19,20,21]. Moreover, in previous studies, amino acid concentrations were mostly determined using amino acid analyzers, but now there exists liquid chromatography–mass spectrometry technology which involves less interference and a higher accuracy [22]. The relationship between macrosomia and amino acids in both maternal and cord sera is rarely reported. This study aims to explore the relationship between macrosomia and amino acids in maternal and cord sera, and we assumed that branched chain amino acids and essential amino acids were significantly correlated with macrosomia.

## 2. Materials and Methods

### 2.1. Ethical Statements

This project has been approved by the Ethics Committee of the Institute of Nutrition and Health, Chinese Center for Disease Control and Prevention (No. 2016-014). All women in the study had provided signed informed consent.

### 2.2. Study Participants

This is a case-control study. A prospective maternal and child nutrition and health cohort in China was selected in Wuqiang, China [23]. Since parity and fetal gender are factors that may affect amino acid levels in maternal and cord sera [24,25], a frequency matching method was adopted to select 39 macrosomia infants (BW ≥ 4000 g) and 39 normal birth weight infants (BW between 2500 g and 3999 g) and their mothers from this cohort. The matching conditions were that the pregnant women were all multipara and the gender ratio of the two groups was similar. These women gave birth between December 2016 and November 2019. The inclusion criteria for pregnant women were: (1) aged 18–45 years old, (2) gestational week > 37, and (3) singleton pregnancy. The exclusion criteria for pregnant women were: (1) foreign nationality, (2) having an infectious disease, (3) history of habitual abortion, and (4) history of diabetes and hypertension and in current pregnancy.

### 2.3. Data Collection

We extracted information from the hospital’s medical record information system, including maternal age, height, weight before pregnancy (self-reported), GWG (pre-delivery weight minus pre-pregnancy weight), gestational age at delivery, mode of delivery, gender, and BW. During the antepartum period, the medical staff were required to collect elbow vein blood from the pregnant women. After the fetus was delivered and the umbilical cord was cut, cord vein blood was extracted. All blood samples were centrifuged at 3500 r/min for 15 min, and the serum was taken and stored at −80 °C until detection. All samples were analyzed for 21 kinds of free amino acids, which include valine (Val), lysine (Lys), leucine (Leu), isoleucine (Ile), phenylalanine (Phe), tryptophan (Trp), threonine (Thr), methionine (Met), histidine (His), glutamate (Glu), glutamine (Gln), asparagine (Asn), aspartic acid (Asp), glycine (Gly), alanine (Ala), tyrosine (Tyr), proline (Pro), ornithine (Orn), arginine (Arg), serine (Ser), and citrulline (Cit). Then the levels of total amino acids (AA_S_), essential amino acids (EAA), NEAA, and branched-chain amino acids (BCAA) were calculated.

### 2.4. Blood Sample Analyses

Amino acids’ levels were measured using liquid chromatography tandem mass spectrometry (LC-MS/MS) (AB SCIEX Triple QuadTM4500, AB Sciex Pte. Ltd., Singapore). The samples were melted at room temperature and mixed evenly by vortex oscillation. A 50 μL sample was added into 100 μL internal standard solution, vortex-mixed for 3 min- The sample was centrifuged at 15,000× *g* for 10 min, and 100 μL supernatant was added into the 96-well sample for LC-MS/MS detection. Analyst software was used to integrate, calculate, and process chromatographic peaks. The ratio of the peak area between the serum and the internal standard was the vertical coordinate (*y*), and the ratio of the concentration between the serum and the internal standard was the horizontal coordinate (*x*). The weighted least square method (weight coefficient was 1/*x^2^*) was used to calculate the concentration of the amino acids. The formula for calculating the concentration of amino acids in serum is as follows: *x = (y − b)/m*, *b* is the intercept of the standard curve, and *m* is the slope of the standard curve.

The quality control methods of amino acids detection were to pass the high concentration quality control products (HQC) and the low concentration quality control products (LQC) through the instrument first. The standard curve was made with the external solution. The detection deviation of HQC was less than 20%, and LQC was less than 15%.

### 2.5. Statistical Analyses

All data were collated and analyzed using SAS 9.4 software (SAS Institute Inc., Cary, NC, USA) and SIMCA 14.1 (Umetrics, Umeaa, Sweden). The Shapiro–Wilk test was used to analyze the normality of quantitative data. Normally distributed data were described with the mean and standard deviation (SD), while non-normally distributed data were described with the median and the interquartile range (IQR). The Student’s *t*-test or the Mann–Whitney U test was used to analyze the differences between the two groups depending on whether it had a normal distribution. Through Orthogonal Partial Least Squares Discriminant Analysis (OPLS-DA), the Variable Importance in Projection (VIP) value was used to analyze the contribution of various amino acids to the differences between the two groups. Unconditional multivariate logistic regression analysis was used to test the association between macrosomia and amino acids in maternal and cord sera. All results with a *p*-value < 0.05 were considered statistically significant.

## 3. Results

### 3.1. Maternal and Neonatal Data

Maternal characteristics included age, pre-pregnancy body mass index (BMI), GWG, parity, and mode of delivery. Neonatal characteristics included gestational age at delivery, gender, birth height, and BW (Table 1). The BW of the macrosomia group was about 800 g higher than that of the control group (*p* < 0.05). The birth length and GWG of the macrosomia group were also higher than that of the control group (*p* < 0.05). There were no significant differences in maternal age, pre-pregnancy BMI, gestational age, gender, and delivery mode between the two groups (*p* > 0.05) (Table 1).

### 3.2. Comparative Analysis of Amino Acids’ Levels in Maternal and Cord Serum between the Two Groups

In maternal serum, the levels of asparagine, glutamine, methionine, alanine, and threonine in the macrosomia group were higher than those in the control group, and the level of arginine was lower than that in the control group (*p* < 0.05). There were no significant differences in other amino acids between the two groups (*p* > 0.05) (Table 2).

In cord serum, the level of glutamate of the macrosomia group was higher than that of the control group (*p* < 0.05), and the levels of lysine, histidine, phenylalanine, arginine, tryptophan, valine, isoleucine, glutamate, tyrosine, BCAA, and EAA were lower than those of the control group (*p* < 0.05), and there were no significant differences in other amino acids between the two groups (*p* > 0.05) (Table 3).

The ratios of EAA, valine, threonine, methionine, tryptophan, and alanine in maternal serum to those in cord serum were higher in the macrosomia group, while the ratio of glutamine was lower in the macrosomia group (Table 4).

**Figure 1:**
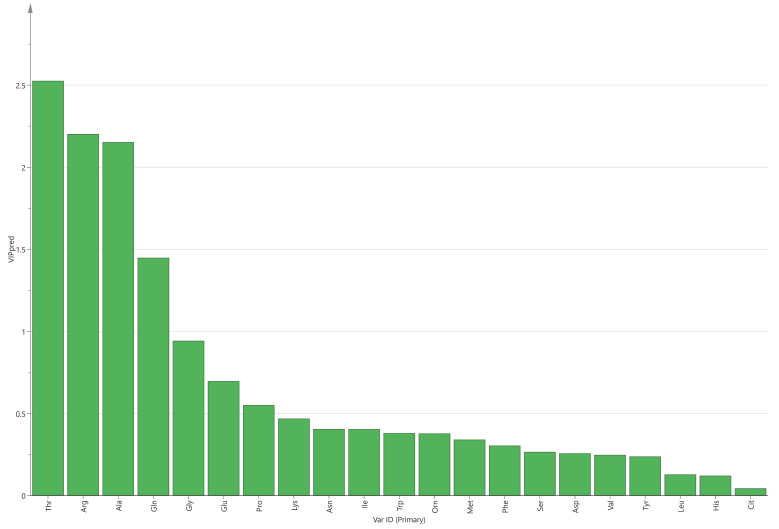
VIP values of amino acids in maternal serum.

**Figure 2:**
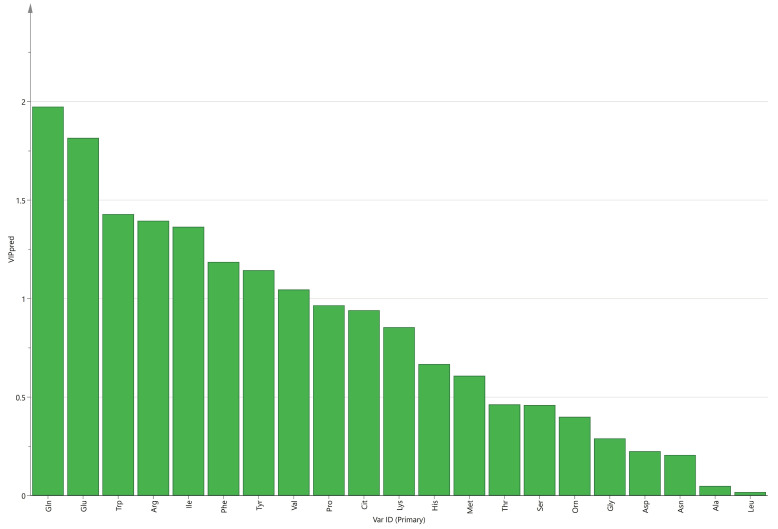
VIP values of amino acids in cord serum.

Through OPLS-DA, the VIP value of each amino acid can be obtained. The amino acids in maternal serum that contributed more to the difference between the two groups were threonine, arginine, alanine, and glutamine (Figure 1). In cord serum, the amino acids that contributed more to the difference between the two groups were glutamine, glutamate, tryptophan, arginine, isoleucine, phenylalanine, tyrosine, and valine (Figure 2).

### 3.3. Relationship between Macrosomia and Amino Acids in Maternal and Cord Serum

The results of the multivariate analysis show that macrosomia is associated with arginine and threonine in maternal serum and associated with glutamine, glutamate, histidine, and isoleucine in cord serum. After adjusting for maternal pre-pregnancy BMI, gestational weight gain, and gestational age, the relationships between macrosomia and these amino acids in maternal and cord sera remained significant (*p* < 0.05), except for isoleucine in cord serum (*p* > 0.05). This means that for every unit (μg/mL) increase in arginine in maternal serum, the risk of macrosomia decreased by 28%, and for every unit increase in threonine, the risk of macrosomia increased by 1.43-fold. In cord serum, the risk of macrosomia was 1.16-fold and 1.12-fold for each unit increase in glutamine and glutamate, respectively, and decreased by 31% for each unit increase in histidine. The ratios of amino acids in maternal serum to those in cord serum were not associated with macrosomia, whether adjusted or not (*p* > 0.05) (Table 5).

## 4. Discussion

This study aimed to analyze the relationship between macrosomia and amino acids in maternal and cord sera. The results show that the levels of asparagine, glutamine, methionine, alanine, and threonine in the maternal sera of the macrosomia group were higher but arginine was lower than that in the control group. The levels of lysine, histidine, phenylalanine, arginine, tryptophan, valine, isoleucine, glutamate, tyrosine, and EAA in the cord sera of the macrosomia group were lower while glutamine was higher than that in the control group. The ratios of EAA, valine, threonine, methionine, tryptophan, and alanine in maternal serum to those in cord serum were higher, while the ratio of glutamine was lower in the macrosomia group.

Fetal growth is a complex and unique process involving the interaction between mother, fetus, and placenta. BW is determined by genetic susceptibility, available nutrients (glucose, lipids, and amount and type of amino acids), and endocrine regulation of the mother, fetus, and placenta [26,27]. The characteristics of this period are rapid growth and development and enormous maternal physiological changes. Fetal growth requires the accumulation of large amounts of protein, synthesized entirely from the cord supply of AAs [28]. We hypothesized that changes in the amino acid content of maternal or cord serum may lead to adverse birth outcomes, such as macrosomia.

### 4.1. Macrosomia and Amino Acids in Maternal Serum

The results show that the levels of asparagine, glutamine, methionine, alanine, and threonine in maternal serum were higher in the macrosomia group, but arginine was lower. Threonine, arginine, alanine, and glutamine in maternal serum contributed more to the difference between the two groups. Multivariate analysis results show that macrosomia is associated with arginine and threonine in maternal serum.

Arginine plays a role in nutritional regulation, enhancing immune function, anti-inflammation, anti-oxidation, regulating lipid metabolism, reducing TC, TG, and LDL-C, and increasing HDL-C [29,30]. Lower levels of arginine in mothers of macrosomia may represent a decline in these functions. Glutamine is a non-essential amino acid and becomes a conditional essential amino acid during pregnancy because fetal demand exceeds maternal synthesis—macrosomia may have higher demand than normal birth weight babies [31]. Threonine is involved in many metabolic processes. One animal study showed that when threonine was deficient, rats gained less weight and expended less energy [32]. Alanine is an important part of the glucose–alanine cycle. Alanine passes through the blood to the liver to synthesize glucose; then glucose is converted to produce alanine. Mothers who delivered macrosomia have higher glucose levels than normal, so the level of alanine in blood was increased [33]. However, explaining how these amino acid changes in the maternal serum affect the formation of macrosomia may require further study of the placental transport mechanism, and these changes may also be the result of the feedback mechanism of macrosomia to the mother.

There are some differences in this study compared to previous studies. Zhang et al. divided BW into three equal parts; they found that compared to the medium birth weight (MBW) group, levels of valine, methionine, isoleucine, leucine, EAA, and BCAA in maternal blood were lower in the high birth weight (HBW) group [34]. Wen et al. found that compared to the normal group, glutamate and phenylalanine in the blood of mothers in the first and second trimesters were higher in the macrosomia group [18]. Moreover, because most of the previous studies focus on the relationship between SGA or IUGR and amino acids in maternal blood, the results also varied widely. For example, Cetin et al. found that the concentrations of phenylalanine, histidine, arginine, alanine, and ornithine in the maternal blood of SGA were higher than those of AGA, but glutamate was lower [21], which may be due to the maladjustment of pregnancy due to insufficient hormone production [35]. However, Tsyvian et al. found that the levels of lysine, leucine, histidine, most of NEAA, and total AA_S_ in the maternal blood of SGA were lower than those of AGA [20]. One study found that the levels of threonine, arginine, glutamate, and taurine in pregnant women with subsequent IUGR were significantly increased in early pregnancy, while the levels of isoleucine, leucine, valine, asparagine, and glutamine were decreased [36]. Moghissi et al. found that maternal glycine, lysine, and total amino acids were positively correlated with BW, while valine and threonine were negatively correlated with BW [37]. Compared to previous studies, there were significant differences between these studies and our study in outcome variables, blood collection time, and results. More research on the correlation between nutrients in maternal blood and macrosomia is needed in the future, which is of great significance for the prevention and treatment of macrosomia.

### 4.2. Macrosomia and Amino Acids in Cord Serum

In this study, the level of glutamine in cord serum of the macrosomia group was higher than that in the control group, and lysine, histidine, phenylalanine, arginine, tryptophan, valine, isoleucine, glutamate, and tyrosine were lower. Glutamine, glutamate, tryptophan, arginine, isoleucine, phenylalanine, tyrosine, and valine contributed significantly to the difference between the two groups. Multivariate analysis results show that macrosomia is associated with glutamate, glutamine, and histidine in cord serum.

Studies on the relationship between amino acids in cord blood and macrosomia are limited. The results of Zhang’s study showed that total EAA, total BCAA, total aromatic amino acid, valine, isoleucine, phenylalanine, lysine, alanine, and homocysteine in the cord blood of HBW were all lower than LBW [34]. This is similar to what we found. One study found that the concentrations of alanine, tyrosine, and most essential amino acids in the cord blood of SGA are lower than those of AGA [20]. Another study found that levels of BCAA, methionine, serine, and tyrosine in the cord blood of SGA were lower than those in AGA [38]. Georgios et al. found that levels of BCAA and alanine in the cord blood of IUGR were higher than those of AGA, but phenylalanine and tryptophan were lower. The interpretation of the high levels of BCAA and alanine is due to the reduced utilization rate of infants [39]. Mansell et al. found that BW was not related to amino acids in cord blood [40]. The outcome variables in these studies are different from those in this study.

Fetal growth and metabolism is an adaptive process and is programmed by intrauterine nutrition and the environment; the placenta can act as a nutritional sensor [41]. If the placenta senses the fetal environment to have low nutrient levels, it increases its transport activity to support normal fetal growth [42]. Similarly, if the placenta perceives a fetal environment with high nutrient levels, it may restrict its transport activities to correct for fetal overgrowth. Moreover, by analyzing the relationship between macrosomia and the ratios of amino acids in maternal serum to those in cord serum, it was found that the ratios of valine, threonine, methionine, tryptophan, and alanine were higher in the macrosomia group, indicating that the transport efficiency of these amino acids by the placenta of macrosomia may be lower. Other studies have reported the mRNA expression of amino acid transporters in system A and the level of SNAT2 (mainly mediating the transport of small neutral amino acids, such as methionine, leucine, and alanine [43]) being negatively associated with BW and the mRNA expression in the placenta of LGA infants being lower [44]. In our study, mothers in the macrosomia group had higher pre-pregnancy BMI compared to the control group (25.1 kg/m^2^ vs. 23.8 kg/m^2^). Farley et al. found that maternal obesity was accompanied by decreased placental SNAT activity [45]. In the in vitro study by Meredith et al., the activity of system A decreased significantly with an increase in amino acid concentrations [46]. Therefore, the explanation for the results is that when the placenta senses that the amino acid level in the cord serum of macrosomia is higher, it adjusts the quantity or activity of transporters to reduce their transport to it [47].

In this study, the association between macrosomia and amino acids in maternal and cord sera was analyzed using a case-control method. This study is a retrospective study, and cannot determine the causal relationship between macrosomia and amino acids in cord blood, and this study did not collect dietary data on pregnant women that could affect amino acid levels. Moreover, there are few supporting data on the correlation between macrosomia and amino acids in maternal or cord serum. In the future, multicentric cohort studies can be conducted to facilitate the determination of causality and predict the occurrence of macrosomia. Future research can also focus on the mechanism of the placenta regulating material transport between mother and fetus and explore the role of the placenta in the formation of macrosomia.

## 5. Conclusions

In summary, amino acids’ levels in maternal and cord sera and ratios of amino acids in maternal serum to those in cord serum were different between macrosomia and normal birth weight newborns. Arginine and threonine in maternal serum and glutamate, glutamine, and histidine in cord serum are closely related to macrosomia.

## Figures and Tables

**Table 1:** Characteristics of study populations.

Characteristics	Macrosomia (*n* = 39)	Control (*n* = 39)	*p*-Value
Maternal age (year)	28.7 ± 3.7	27.8 ± 2.5	0.203
Pre-pregnancy BMI (kg/m^2^)	25.1 ± 4.1	23.8 ± 4.5	0.084
GWG (kg)	16.3 ± 3.1	15.0 ± 2.9	0.049
Parity	2.3 ± 0.7	2.1 ± 0.4	0.226
Cesarean section (%)	53.9	48.7	0.651
Gestational age at delivery (week)	40.1 ± 1.0	39.6 ± 1.2	0.071
Birth weight (g)	4239.7 ± 172.9	3402.6 ± 309.3	<0.001
Birth length (cm)	51.9 ± 1.1	50.0 ± 0.2	<0.001
Gender (boy, %)	51.3	51.3	1.000

BMI, body mass index; GWG, gestational weight gain.

**Table 2:** Comparison of maternal amino acids’ levels between the two groups (Mean ± SD or Median and IQR).

Amino Acid (μg/mL)	Macrosomia (*n* = 39)	Control (*n* = 39)	*p*-Value
Total AAs	325.88 (302.99, 380.30)	318.54 (289.43, 379.70)	0.108
EAA	136.67 ± 23.09	127.73 ± 31.37	0.118
BCAA	44.48 ± 7.93	43.24 ± 11.87	0.560
NEAA	205.93 ± 30.24	200.43 ± 41.69	0.423
Val	21.97 ± 3.67	20.87 ± 5.52	0.303
Leu	14.59 ± 3.30	13.71 ± 4.17	0.308
Ile	7.92 ± 1.66	8.66 ± 2.60	0.139
Thr	27.10 (22.60, 28.90)	19.30 (17.10, 21.80)	<0.001
Lys	19.90 (17.60, 26.80)	18.60 (16.70, 23.50)	0.356
Met	3.70 ± 0.80	3.17 ± 0.83	0.006
His	15.27 ± 3.23	15.35 ± 4.00	0.921
Phe	14.99 ± 3.21	15.08 ± 3.71	0.963
Trp	9.55 ± 1.34	9.70 ± 2.72	0.965
Asn	6.63 ± 1.57	5.88 ± 1.76	0.037
Asp	5.77 ± 1.88	6.02 ± 2.11	0.599
Gln	48.90 (44.60, 54.00)	45.30 (39.80, 50.90)	0.045
Glu	14.13 ± 5.99	16.01 ± 6.63	0.177
Arg	19.74 ± 5.36	25.28 ± 7.00	<0.001
Cit	3.22 ± 0.78	3.31 ± 1.18	0.694
Tyr	9.99 ± 2.11	10.16 ± 2.82	0.760
Orn	6.29 (5.19, 8.03)	5.74 (4.26, 7.24)	0.148
Gly	17.30 (7.50, 19.70)	15.80 (7.50, 18.30)	0.103
Ala	36.40 (33.60, 39.50)	31.70 (28.10, 34.80)	<0.001
Ser	15.50 (11.70, 19.00)	16.00 (13.70, 18.10	0.500
Pro	18.80 (17.20, 23.90)	18.70 (14.60, 21.70)	0.335

AA_S_, amino acids; EAA, essential amino acid; BCAA, branched chain amino acid; NEAA, non-essential amino acid; Val, valine; Thr, threonine; Leu, leucine; Ile, isoleucine; Lys, lysine; Met, methionine; His, histidine; Phe, phenylalanine; Trp, tryptophan; Asn, asparagine; Asp, aspartic acid; Gln, glutamine; Glu, glutamate; Arg, arginine; Cit, citrulline; Tyr, tyrosine; Orn, ornithine; Gly, glycine; Ala, alanine; Ser, serine; Pro, proline.

**Table 3:** Comparison of amino acid levels in cord serum between the two groups (Mean ± SD or Median and IQR).

Amino Acid (μg/mL)	Macrosomia (*n* = 39)	Control (*n* = 39)	*p*-Value
Total AAs	423.60 (391.89, 460.66)	422.07 (375.95, 542.53)	0.589
EAA	183.20 ± 25.55	200.97 ± 48.21	0.047
BCAA	56.60 ± 9.23	62.77 ± 15.66	0.037
NEAA	238.56 (225.49, 255.61)	233.05 (210.77, 299.85)	0.758
Val	29.15 ± 5.13	33.82 ± 8.81	0.006
Leu	17.80 (15.50, 19.00)	15.30 (12.80, 19.00)	0.133
Ile	10.32 ± 1.93	12.50 ± 3.14	<0.001
Thr	31.52 ± 7.59	29.95 ± 8.90	0.253
Lys	43.00 ± 7.93	49.33 ± 14.43	0.020
Met	4.80 (4.44, 5.16)	5.04 (4.17, 6.19)	0.366
His	17.52 ± 2.59	19.24 ± 4.54	0.045
Phe	15.90 (14.20, 17.60)	17.70 (14.80, 21.60)	0.019
Trp	15.50 (14.40, 16.70)	16.50 (14.50, 22.40)	0.022
Asn	6.16 ± 1.57	5.96 ± 1.73	0.500
Asp	4.13 (3.47, 4.85)	4.51 (3.41, 5.63)	0.512
Gln	55.60 (37.90, 65.50)	33.70 (20.60, 46.30)	<0.001
Glu	31.02 ± 17.48	45.38 ± 19.34	<0.001
Arg	11.67 ± 4.31	13.85 ± 5.16	0.046
Cit	2.80 (2.49, 3.22)	2.94 (2.48, 3.40)	0.256
Tyr	12.50 (11.90, 15.30)	14.40 (12.20, 18.40)	0.048
Orn	18.74 ± 5.48	21.07 ± 8.61	0.255
Gly	22.60 (20.00, 25.40)	22.70 (19.50, 29.10)	0.601
Ala	40.30 ± 6.76	41.28 ± 10.27	0.727
Ser	18.66 ± 3.08	20.24 ± 5.38	0.182
Pro	20.50 (17.60, 22.30)	19.70 (17.00, 23.90)	0.948

AA_S_, amino acids; EAA, essential amino acid; BCAA, branched chain amino acid; NEAA, non-essential amino acid; Val, valine; Thr, threonine; Leu, leucine; Ile, isoleucine; Lys, lysine; Met, methionine; His, histidine; Phe, phenylalanine; Trp, tryptophan; Asn, asparagine; Asp, aspartic acid; Gln, glutamine; Glu, glutamate; Arg, arginine; Cit, citrulline; Tyr, tyrosine; Orn, ornithine; Gly, glycine; Ala, alanine; Ser, serine; Pro, proline.

**Table 4:** Comparison of ratios of maternal serum to cord serum amino acids between the two groups (Mean ± SD or Median and IQR).

Maternal/Cord	Macrosomia (*n* = 39)	Control (*n* = 39)	*p*-Value
Total AAs	0.78 (0.71, 0.93)	0.73 (0.59, 0.90)	0.194
EAA	0.72 (0.64, 0.83)	0.62 (0.51, 0.77)	0.027
BCAA	0.77 (0.66, 0.87)	0.71(0.52, 0.87)	0.088
NEAA	0.85 (0.75, 0.99)	0.85(0.65, 1.00)	0.596
Val	0.73 (0.65, 0.86)	0.62 (0.47, 0.78)	0.004
Leu	0.82 (0.69, 1.04)	0.90(0.63, 1.15)	0.826
Ile	0.75 (0.62, 0.85)	0.72(0.56, 0.89)	0.225
Thr	0.89 (0.76, 0.93)	0.69 (0.59, 0.80)	<0.001
Lys	0.48 (0.38, 0.64)	0.39 (0.30, 0.55)	0.051
Met	0.73 (0.61, 0.98)	0.57 (0.42, 0.85)	0.008
His	0.89 (0.76, 0.98)	0.78 (0.61, 1.08)	0.191
Phe	0.95 ± 0.27	0.85 ± 0.29	0.107
Trp	0.60 (0.56, 0.68)	0.51 (0.39, 0.68)	0.032
Asn	1.03 (0.86, 1.39)	1.06 (0.70, 1.29)	0.285
Asp	1.36(0.98, 1.88)	1.46 (0.84, 1.99)	0.818
Gln	0.88 (0.78, 1.23)	1.40(1.03, 2.18)	0.004
Glu	0.44 (0.31, 0.77)	0.37 (0.25, 0.49)	0.077
Arg	1.77 (1.23, 2.27)	1.81 (1.31, 2.73)	0.772
Cit	1.18 ± 0.33	1.10 ± 0.43	0.375
Tyr	0.71 (0.65, 0.87)	0.62 (0.51, 0.88)	0.088
Orn	0.36 (0.28, 0.48)	0.30 (0.19, 0.42)	0.089
Gly	0.79 (0.53, 0.93)	0.58 (0.35, 0.86)	0.054
Ala	0.96 ± 0.24	0.81 ± 0.21	0.004
Ser	0.86 ± 0.27	0.85 ± 0.29	0.931
Pro	1.02 (0.81, 1.12)	0.90 (0.78, 1.14)	0.516

AA_S_, amino acids; EAA, essential amino acid; BCAA, branched chain amino acid; NEAA, non-essential amino acid; Val, valine; Thr, threonine; Leu, leucine; Ile, isoleucine; Lys, lysine; Met, methionine; His, histidine; Phe, phenylalanine; Trp, tryptophan; Asn, asparagine; Asp, aspartic acid; Gln, glutamine; Glu, glutamate; Arg, arginine; Cit, citrulline; Tyr, tyrosine; Orn, ornithine; Gly, glycine; Ala, alanine; Ser, serine; Pro, proline.

**Table 5:** Multivariate analysis of the association between macrosomia and amino acids in maternal and cord sera.

Amino Acid	OR	95% CI	*p*-Value	Adjusted OR ^#^	95% CI	*p*-Value
Maternal serum						
Asn	0.74	0.38–1.44	0.371	0.73	0.35–1.53	0.408
Met	2.55	0.70–9.22	0.154	3.84	0.95–15.62	0.060
Arg	0.76	0.65–0.87	<0.001	0.72	0.60–0.86	0.000
Gly	1.04	0.90–1.20	0.610	1.02	0.84–1.23	0.859
Ala	1.05	0.90–1.22	0.561	1.04	0.87–1.25	0.637
Thr	1.31	1.11–1.55	0.002	1.43	1.15–1.78	0.001
Cord serum						
Lys	1.00	0.91–1.11	0.999	1.02	0.90–1.15	0.786
Glu	1.14	1.04–1.25	0.004	1.12	1.02–1.23	0.015
Gln	1.19	1.09–1.31	0.000	1.16	1.06–1.28	0.002
Met	0.81	0.38–1.72	0.587	1.05	0.46–2.42	0.901
His	0.67	0.48–0.92	0.015	0.69	0.48–0.99	0.047
Phe	1.10	0.86–1.41	0.434	1.07	0.82–1.40	0.614
Arg	1.05	0.90–1.22	0.541	1.05	0.90–1.23	0.535
Trp	0.69	0.47–1.03	0.071	0.71	0.46–1.08	0.105
Tyr	1.23	0.81–1.87	0.342	1.12	0.71–1.77	0.613
Val	0.94	0.76–1.15	0.532	0.94	0.75–1.19	0.623
Ile	0.66	0.44–0.99	0.047	0.65	0.42–1.00	0.051
Maternal/cord						
Val	2.78	0.02–326.30	0.674	4.76	0.04–637.65	0.532
Thr	36.99	0.50–999.99	0.100	54.72	0.39–999.99	0.114
Met	0.68	0.01–49.92	0.858	0.72	0.01–89.36	0.895
Trp	0.16	0.01–10.67	0.392	0.31	0.01–23.79	0.597
Gln	0.58	0.33–1.02	0.059	0.65	0.37–1.15	0.137
Ala	8.19	0.20–337.06	0.267	1.80	0.02–155.33	0.796

# gestational age, pre-pregnancy BMI, and gestational weight gain were adjusted.

## Data Availability

The datasets generated or analyzed during the current study are not publicly available due to the data management requirements of our institution but are available from the corresponding author upon reasonable request.
